# Endurance Training Improves Insulin Sensitivity, Metabolic Flexibility, and Mitochondrial Function in Type 2 Diabetes

**DOI:** 10.1111/sms.70383

**Published:** 2026-10-24

**Authors:** Maria Hansen, Sophie Møller, Ida Blom, Kristine K. Lange, Andreas K. Ziegler, Johanne E. Farlov, Jacob Borch, Sofie A. Hesselager, Jonathan K. Hansen, Anna Liebmann, Mette H. Dal, Thorbjörn Åkerström, Jørn W. Helge, Ylva Hellsten, Flemming Dela

**Affiliations:** ^1^ Department of Biomedical Sciences, Faculty of Health and Medical Sciences University of Copenhagen Copenhagen Denmark; ^2^ The August Krogh Section for Human Physiology, Department of Nutrition, Exercise and Sports, Faculty of Science University of Copenhagen Copenhagen Denmark; ^3^ Diabetes Pharmacology Novo Nordisk A/S Måløv Denmark

**Keywords:** hyperinsulinaemic clamp, metabolic flexibility, mitochondrial respiratory capacity, non‐randomized study, peak fat oxidation, reactive oxygen species

## Abstract

To determine how endurance training influences insulin sensitivity, metabolic flexibility, and mitochondrial function in individuals with and without type 2 diabetes (T2D). Insulin sensitivity (2‐step hyperinsulinaemic–isoglycaemic clamp), metabolic flexibility, and mitochondrial respiratory capacity and H_2_O_2_ emission were assessed in participants with T2D (*n* = 15) and controls (*n* = 14) before and after 12 weeks of supervised endurance training. Sensitivity analyses were performed using linear mixed‐effects models including all available participants. At baseline, glucose clearance was lower in individuals with T2D compared with controls. Training increased glucose clearance (mL/min/kg FFM) in both groups, with larger model‐derived improvements in T2D (Step 1: +1.43 [0.88–1.97]; Step 2: +3.18 [2.12–4.23]) than in controls (Step 1: +0.76 [0.21–1.31]; Step 2: +1.49 [0.53–2.45]). The group × time interaction was significant at the higher insulin step. Metabolic flexibility to fat—but not glucose—was reduced in T2D and improved modestly with training. Mitochondrial respiratory capacity did not differ between groups; however, training decreased mitochondria‐specific respiration and H_2_O_2_ emission normalized to CS activity and respiration. Endurance training enhances insulin sensitivity and fat oxidation in individuals with T2D and induces favorable mitochondrial adaptations, despite the absence of intrinsic mitochondrial dysfunction.

## 1 Introduction

The prevalence of type 2 diabetes (T2D) is steadily increasing worldwide, and a major characteristic, insulin resistance, has been associated with metabolic inflexibility and mitochondrial dysfunction [1, 2, 3, 4], although studies have questioned these relationships [5, 6, 7, 8, 9, 10].

Metabolic flexibility is the ability to adapt to changes in substrate availability, an important function of the mitochondria. It is traditionally assessed by the change in respiratory exchange ratio (RER) in response to a hyperinsulinaemic clamp [11, 12], but has in recent years expanded to include other metabolic challenges, like fat oxidation capacity during a graded exercise test [5, 13]. However, it is unclear whether individuals with and without T2D have different peak fat oxidation (PFO) rates [14, 15, 16]. While a positive correlation between insulin sensitivity and metabolic flexibility has been found in individuals with and without T2D, other studies show mixed results, as shown in a recent review [17].

Exercise training improves insulin sensitivity in T2DM [18], metabolic flexibility [1, 14, 19, 20] and mitochondrial respiratory capacity [1, 3, 8], with the latter possibly reflecting increased mitochondrial content.

Here, we investigated insulin sensitivity, metabolic flexibility to glucose (clamp) and fat (PFO), along with mitochondrial respiratory capacity and H_2_O_2_ emission in T2D and in response to endurance training. We hypothesized that insulin sensitivity and metabolic flexibility would be lower in the diabetes group, and that training would improve both parameters. Additionally, we hypothesized similar absolute and relative (mass‐ and mitochondria‐specific, respectively) respiration but higher H_2_O_2_ emission in T2D, as well as increased absolute but decreased relative respiration and H_2_O_2_ emission after training.

To our knowledge, this is the first study to simultaneously assess insulin sensitivity, metabolic flexibility to both glucose and fat substrates, and mitochondrial function—including respiratory capacity and H_2_O_2_ emission—in individuals with T2D before and after endurance training using state‐of‐the‐art methodologies. While previous studies have investigated insulin sensitivity using hyperinsulinaemic euglycaemic clamps and mitochondrial respiration in isolated cell types, our study uniquely integrates whole‐body substrate utilization (via hyperinsulinaemic, iso‐glycaemic clamp and PFO testing) with direct measures of mitochondrial respiratory capacity and ROS production ex vivo. Furthermore, by evaluating both mass‐specific and mitochondria‐specific respiration, we distinguish between changes in mitochondrial quantity and intrinsic function—an approach not commonly applied in T2D exercise studies. This design enables a more nuanced understanding of the interplay between insulin resistance, metabolic flexibility, and mitochondrial dynamics in T2D, and how these adapt to endurance training.

## 2 Methods

This non‐randomized, two‐group intervention study was reported following the TREND statement for transparent reporting of non‐randomized evaluations, as recommended by the EQUATOR Network (Figure S1); the completed checklist is available in the Supporting Information (Table S1). All participants gave written informed consent before participation, and the study was performed according to the guidelines of the Declaration of Helsinki, approved by the Regional Scientific Ethical Committees for the Capital Region of Denmark (H‐20046605), and registered at ClinicalTrials.gov (NCT04945551).

### 2.1 Study Population

We enrolled 20 individuals with T2D and 17 without, matched for age and BMI. Groups were matched for BMI to minimize the confounding effects of adiposity and isolate the impact of T2D. The participants were recruited through social media. The unit of assignment was the individual participant, who was allocated to either the T2D or control group based on diabetes status. Participants were sedentary (no regular exercise), had no metabolic (besides T2D), kidney, or major cardiovascular diseases (mild‐to‐moderate hypertension, hypercholesterolaemia, and asthma were accepted), and had normal resting ECGs. Glucose‐lowering medications other than insulin were allowed, and diabetes was defined as HbA_1c_ > 47 mmol/mol regardless of treatment. In the T2D group, glucose‐lowering therapy included metformin (*n* = 12), SGLT2 inhibitor (*n* = 1), and GLP‐1 receptor agonist (*n* = 2). Antihypertensive treatment in T2D comprised ACE inhibitors (*n* = 5), ATII receptor antagonists (*n* = 2), calcium channel blockers (*n* = 2), and diuretics (*n* = 1), either as monotherapy or in combination. In the control group, one participant was treated with an ACE inhibitor and one with an ATII receptor antagonist; no participants used calcium channel blockers. Cholesterol‐lowering therapy included statins (T2D *n* = 7; CON *n* = 2) and one cholesterol absorption inhibitor (T2D *n* = 1). The medication for each participant remained unchanged throughout the study period. All participants gave written informed consent before participation. Five participants in the T2D group and three in the control group did not complete the full study due to illness or lack of time. Participants with incomplete pre/post data did not contribute to complete‐case repeated‐measures analyses, whereas all available observations were retained in the linear mixed‐effects model analyses.

### 2.2 Experimental Protocol

Participant recruitment, testing, and intervention were conducted throughout the period from January 2021 to December 2023. All tests and training took place at the Panum Building, University of Copenhagen, Denmark.

Participants arrived at the laboratory at 8 a.m. after a 10‐h overnight fast (including medication pause) on two test days separated by at least 48 h and held within 1 week of each other. The post‐intervention test days were planned so that both test days occurred 40–48 h after a training session (Figure 1). Participants were asked to abstain from moderate to vigorous physical activity 48 h before the two test days and to follow a three‐day diet plan consuming ≥ 250 g of carbohydrate/day.

**FIGURE 1:**
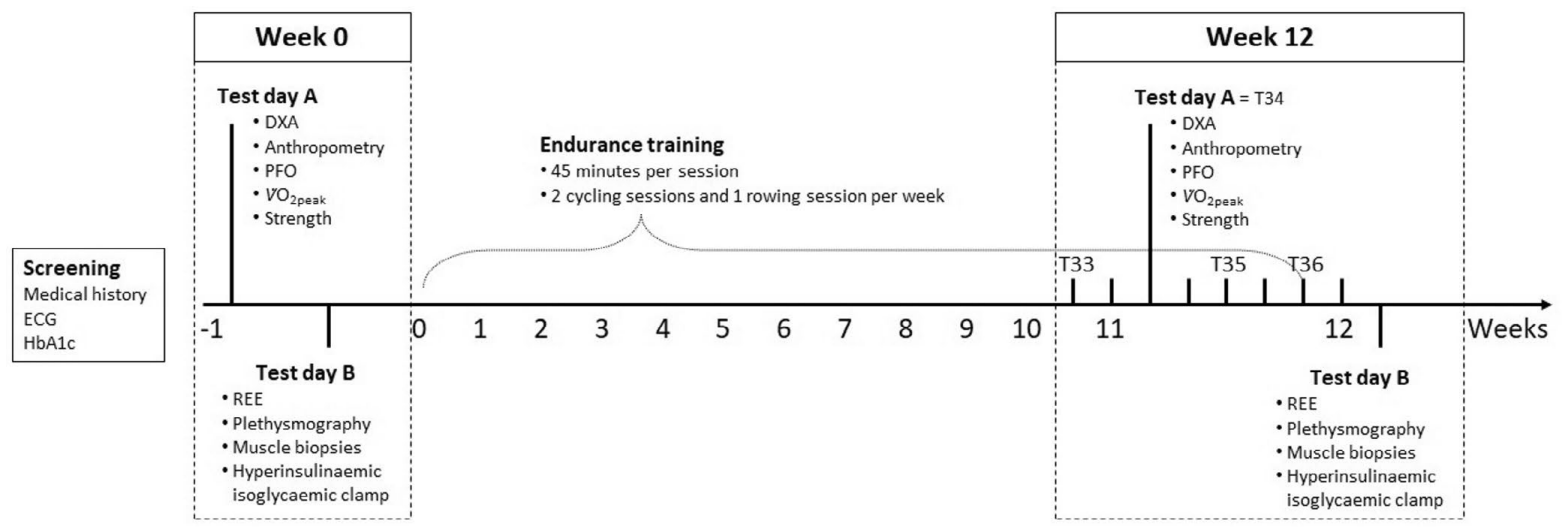
Study overview. DXA, dual‐energy X‐ray absorptiometry; PFO, peak fat oxidation, REE, resting energy expenditure; T33–T36, the last four training days in the intervention.

### 2.3 Exercise Intervention

The intervention consisted of 12 weeks of supervised 45‐min endurance training sessions on a cycle (2 sessions/week; Lode Corrival, Lode BV, Netherlands) and a rowing ergometer (1 session/week; Concept 2 RowErg, USA). The two different cycling programmes used consisted of intervals and were designed from the *V̇*O_2peak_ test to reach an average of 70% (range 55%–80%) of the *V̇*O_2_ reserve [*V̇*O_2peak_–*V̇*O_2rest_]. Cycling workload was prescribed from individual graded *V̇*O_2peak_ tests to achieve the target %*V̇*O_2_ reserve, with HR_rest_ measured at rest and HR_max_ obtained during the *V̇*O_2peak_ test for calculation of heart rate reserve. The cycling sessions consisted of structured interval blocks (typically 3–6 intervals of moderate‐to‐high intensity interspersed with active recovery), with intensity individually adjusted to maintain an average of ~70% *V̇*O_2_ reserve (corresponding to ~70% heart rate reserve). Workload was progressively increased across the 12‐week intervention based on repeated *V̇*O_2peak_ testing (every three weeks) and continuous heart rate monitoring to ensure maintenance of the intended relative intensity. The intervention aimed to induce a consistent improvement in aerobic fitness rather than to compare specific interval configurations or dose–response effects.

During rowing, the training programme was designed for the participant to reach an average of 70% of their heart rate reserve, and the supervisor adjusted the load (cycling) or strokes per minute (rowing) if necessary. Heart rate was monitored throughout all sessions.

### 2.4 Test Day A

#### 2.4.1 Body Composition and Anthropometry

We performed a whole‐body dual X‐ray absorptiometry (DXA) scan (Lunar Prodigy Advance, iDXA, GE Healthcare, USA) after the participant had emptied their bladder. The software program Corescan determined visceral fat content. Following the scan, we measured blood pressure in the supine position, and waist, hip, and thigh circumference.

#### 2.4.2 Cycling Tests

We examined substrate utilization during exercise as a measure of metabolic flexibility, including PFO, using breath‐by‐breath indirect calorimetry (Quark CPET, COSMED, Italy) during a graded exercise test on a cycle ergometer (Monark 839E, Monark Exercise AB, Sweden). We used the protocol validated in individuals with obesity by Dandanell et al. [21] with minor changes. In brief, the warm‐up period was five minutes at 30 W, followed by increases in workload by 20 W (women) or 25 W (men) every three minutes. After five minutes of rest, a *V̇*O_2peak_ test began. Women and men started at 60 and 75 W, respectively, and the load increased every minute by 20 and 25 W, respectively, until voluntary exhaustion and RER values > 1.10.

#### 2.4.3 Strength and Functional Performance

We measured handgrip strength three times on each side (one minute in between) with a handheld dynamometer (Takei Grip‐D T.K.K.5401, Japan). Leg power was measured in each leg by the Nottingham Power Rig until two measurements were below the highest obtained value. Finally, participants performed a 30‐s sit‐to‐stand test.

### 2.5 Test Day B

#### 2.5.1 Hyperinsulinaemic–Isoglycaemic Clamp

An arterial catheter was inserted into a radial or brachial artery and peripheral venous catheters into an antecubital vein of each arm for arterial–venous blood‐sampling and insulin and glucose infusions during a two‐step (40 and 400 mU/m^2^/min, 2 h each) hyperinsulinaemic–isoglycaemic clamp (i.e., plasma glucose was kept at the individual's fasting level). A 2 mL bolus of insulin infusate (mixed insulin [Actrapid 100 IE/mL, Novo Nordisk, Denmark], 2.5 mL plasma, and saline in a 50 mL syringe) initiated the clamp, plasma glucose was analyzed every five minutes (ABL 800Flex, Radiometer, Denmark), and a 20% glucose infusion was adjusted to keep the plasma glucose stable. Arterial and venous blood were sampled in the basal state and during the final 20 min of each clamp step. The glucose infusion rate was averaged over the final 30 min of assumed steady state and corrected for urinary glucose loss. To account for plasma glucose differences, glucose clearance [glucose infusion rate/plasma glucose] was used to measure insulin sensitivity.

#### 2.5.2 Resting Energy Expenditure and Substrate Use

We used the ventilated hood method (Q‐NRG, COSMED, Italy) in a supine position for 20 min in the basal state and during steady state of each clamp step. The software program Omnia selected data from a five‐minute period of lowest variability in energy expenditure for analysis. We assumed 16% nitrogen content in protein and calculated protein turnover from urine urea content. Only carbohydrate and lipid metabolism were assumed to contribute to nonprotein *V̇*O_2_ [*V̇*O_2total_–*V̇*O_2protein_], with *V̇*O_2protein_ being calculated from known values of O_2_ turnover (0.97 L/g protein). Correspondingly, *V̇*CO_2_ was calculated (0.77 L CO_2_ produced/g protein). O_2_ turnover and CO_2_ production for glycogen (basal state; 0.828 L/g for both), glucose (during the clamp; 0.746 L/g for both), and lipid (2.03 and 1.43 L/g, respectively) were used to calculate the glucose and lipid oxidation rates. Non‐oxidative glucose disposal was estimated as [glucose infusion rate–glucose oxidation]. The change in RER from basal to each clamp step reflects metabolic flexibility to glucose. When RER approaches or exceeds 1.0, the stoichiometric calculation may yield zero or negative estimates of lipid oxidation. Such estimates should be interpreted as essentially complete suppression of net lipid oxidation under these conditions rather than as literal “negative” fat oxidation.

#### 2.5.3 Forearm Venous Occlusion Plethysmography

An indium‐gallium strain‐gauge (D.E. Hokanson, USA), ~2 cm shorter than the circumference, was placed around the widest part of the participant's forearm and connected to a plethysmograph (EC6, Hokanson). With the participant supine, an inflation system (E20 rapid cuff inflator and AG101 cuff inflator air source, Hokanson) inflated a cuff on the upper arm five times for 8 s to 40 mmHg, with 12 s between measurements to allow venous emptying. The RS232 digital output connected to NIVP3 Software (Hokanson) for documentation and automatic control of the rapid cuff inflation system. The forearm was placed at an incline on a custom‐made pillow with an indentation to prevent interference with the strain‐gauge. After the measurements were obtained, we drew a line in the NIVP3 software program to match the slope of each measurement. The program calculated this slope with the unit %/minute which corresponded to the inflow of arterial blood in the forearm.

#### 2.5.4 Muscle Biopsies

Lateral vastus muscle biopsies were obtained after injection of subcutaneous local anesthesia (Xylocain 20 mg/mL, Aspen Pharma Trading Limited, Ireland). Macroscopic connective tissue and blood were immediately removed, and part of the muscle tissue was kept in ice‐cold preservation buffer (BIOPS [22]) to prepare for imminent respirometry and fluorometry. The rest was snap‐frozen in liquid nitrogen and stored at −80°C until further analysis.

### 2.6 Plasma Analyses

Spectrophotometry (Cobas 6000c 501, Roche, Denmark) was used to analyze plasma concentrations of cholesterol (total, HDL, and LDL), triglycerides, hsCRP, insulin, C‐peptide, growth hormone, cortisol, ketone bodies, glycerol, and NEFA. Adiponectin was analyzed by ELISA. HbA_1c_ was measured on a DCA Vantage Analyzer (Siemens Healthcare Diagnostics, USA).

### 2.7 Muscle Analyses

#### 2.7.1 Mitochondrial Respiration

All respiratory and H_2_O_2_ emission measurements were made in duplicate in mitochondrial respiration medium (MIR05 [6]) in a hyper‐oxygenated environment ([O_2_] 200–450 nmol/mL). Skeletal muscle fibers were prepared by permeabilization with saponin [50 μg/mL] for 30 min in BIOPS followed by a 2 × 10 min wash in ice‐cold MIR05. Mitochondrial respiration (normalized to mg wet weight) was measured using high‐resolution respirometry (Oxygraph‐2 k, Oroboros Instruments, Innsbruck, Austria) as previously described [6]. In short, leak respiration with electron input of complex I (CI_leak_) was measured by adding malate [2 mmol/L], glutamate [10 mmol/L], and pyruvate [5 mmol/L], followed by cytochrome *c* [10 μmol/L] to evaluate outer membrane integrity, which in all cases was undisturbed. To assess oxidative phosphorylation capacity with electron input of complex I, ADP was added [5 mmol/L], accompanied by MgCl_2_ [3 mmol/L] (CI_p_). Next, succinate [10 mmol/L] was added to measure oxidative phosphorylation capacity with simultaneous electron input of complex I and II (CI + II_p_). Finally, carbonyl cyanide‐4‐(trifluoromethoxy) phenylhydrazone (FCCP) was titrated in stepwise increments until maximum uncoupling was reached (ETC).

#### 2.7.2 Mitochondrial H_2_O_2_ Emission

H_2_O_2_ emission was measured via fluorometry, using the H_2_O_2_‐sensitive probe Amplex UltraRed, which reacts with H_2_O_2_ in the presence of horseradish peroxidase to produce the highly stable fluorophore resorufin. As H_2_O_2_ emission measurements are oxygen‐dependent, the measurements were conducted at approximately the same oxygen concentration. The protocol was initiated by addition of blebbistatin [5 μmol/L] to inhibit muscle contraction, superoxide dismutase [90 U/mL] to convert O_2_^•−^ to H_2_O_2_, and Amplex UltraRed [20 μmol/L], after which baseline H_2_O_2_ emission was determined by addition of horseradish peroxidase [2 U/mL]. H_2_O_2_ emission of complex I was determined after addition of malate [2 mmol/L] and pyruvate [5 mmol/L] (CI_leak_). Then, a three‐step succinate titration [1 mmol/L, 2 mmol/L, 3 mmol/L] was performed to measure maximal H_2_O_2_ emission of complexes I and II (CI + II_leak_). 0.1 μmol/L H_2_O_2_ was added after each step to correct for any changes in assay sensitivity.

#### 2.7.3 Citrate Synthase (CS) and Hydroxyacyl‐CoA Dehydrogenase (HAD)

CS and HAD activities were measured as previously described [23, 24]. Freeze‐dried muscle (range 1.36–4.15 mg) dissected free of blood, adipocytes, and connective tissue was used for the assay. Enzyme activity was determined as μmol substrate/min/g dry weight muscle. Mitochondria‐specific respiration was normalized to CS activity.

### 2.8 Statistical Analyses

The primary outcome was insulin sensitivity (glucose clearance from the clamp), and secondary outcomes were muscle strength, *V̇*O_2peak_, PFO, mitochondrial respiratory capacity, and ROS production. Statistical analyses were performed using RStudio (ver. 2023.06.1 + 524; RStudio, USA). Residual, Q–Q plots, and Shapiro–Wilk tests were used to assess normality and homogeneity of variance. For outcomes not normally distributed, either log‐ or Box–Cox transformations were applied as appropriate.

The primary outcome, glucose clearance, was initially analyzed using repeated‐measures ANOVA in participants with complete pre‐ and post‐intervention data. To utilize all available observations and to assess the robustness of the complete‐case findings, complementary linear mixed‐effects models (LMMs) were subsequently fitted with subject included as a random effect. The LMM approach accounts for within‐subject correlation and provides valid inference under a missing‐at‐random assumption. For secondary outcomes, main effects and interactions were examined using two‐way repeated‐measures ANOVA based on participants with complete pre‐ and post‐intervention data. In the case of significant interactions, post hoc comparisons were performed using LMMs with Tukey‐adjusted tests and false discovery rate (FDR) correction. Group, Training (Pre vs. Post), and clamp or respiration state were included as fixed variables where applicable. Figures were prepared using GraphPad Prism 10.2.3. All results are reported as mean ± SD unless otherwise specified, and *p* < 0.05 was considered statistically significant.

To quantify the achieved power for the primary endpoint, we performed a post hoc power analysis based on the exact noncentral‐*t* distribution, which accounts for finite sample sizes and the observed pre–post correlations (Table S2). To include all available participants, including those with incomplete pre/post data, we additionally fitted linear mixed‐effects models with subject ID as a random intercept (Table S3). For each clamp step, glucose clearance was modeled as a function of Group, Training, and their interaction, and estimates are reported as Estimate (95% CI), *p*‐value. This approach provides a valid inference under a missing‐at‐random assumption and avoids listwise deletion. A combined model including clamp Step and all two‐ and three‐way interactions was also fitted as a sensitivity analysis. Effect sizes are reported as Cohen's *dz* (within‐group paired changes) and Hedges' *g* (between‐group differences in change), with 95% confidence intervals. The unit of analysis was the individual participant, consistent with the unit of assignment.

Investigators were not blinded to group allocation, as diabetes status was evident during testing and glucose infusion rates were visible in real time. To reduce bias, however, research staff did not access or recall participants' pre‐training infusion rates when performing the post‐training clamps, which were conducted approximately 3 months later.

No subgroup analyses were prespecified beyond the comparisons of T2D and control groups.

## 3 Results

### 3.1 Participant Characteristics

Fifteen participants (ten men, five women) with T2D and 14 control participants (nine men, five women) finished 12 weeks of endurance training. The general characteristics of the participants are presented in Table 1. The groups had similar bodyweight and body composition; however, the diabetes group had higher HbA_1c_ and lower total cholesterol and LDL‐cholesterol than the control group (for all: *p* < 0.001). No changes in body composition or plasma biochemical characteristics were observed from pre‐ to post‐training. No serious adverse events related to the training intervention or experimental procedures were observed.

**TABLE 1:** Participant characteristics at baseline and after training.

	CON		T2D		Main effect		Interaction (group × training)
	Pre	Post	Pre	Post	Group	Training	
Sex (male/female)	9/5	9/5	10/5	10/5			
Age (years)	54.6 ± 7.5		52.7 ± 6.6		0.46^a^		
Time since diagnosis (years)			4.1 ± 2.5				
Body mass (kg)	106 ± 16	104 ± 17	103 ± 19	102 ± 19	0.62	0.78	0.98
BMI (kg/m^2^)	33.9 ± 4.4	33.4 ± 4.3	32.2 ± 3.7	31.9 ± 3.7	0.14	0.64	0.99
Systolic BP (mmHg)	141 ± 17	141 ± 13	138 ± 21	140 ± 19	0.71	0.83	0.81
Diastolic BP (mmHg)	92 ± 10	90 ± 9	91 ± 14	93 ± 11	0.80	0.88	0.46
**Body composition**							
Whole‐body fat (%)	39.0 ± 5.0	37.6 ± 5.0	38.5 ± 5.9	37.3 ± 5.8	0.79	0.38	0.95
Whole‐body fat (kg)	40.7 ± 5.5	38.7 ± 5.4	39.4 ± 8.1	37.7 ± 8.0	0.53	0.32	0.93
VAT (kg)	2.5 ± 1.0	2.4 ± 0.9	3.0 ± 1.3	2.7 ± 1.2	0.15	0.41	0.42
Whole‐body FFM (kg)	64.9 ± 13.3	65.5 ± 13.9	63.8 ± 14.4	64.3 ± 14.6	0.38	0.85	0.84
Abdominal circumference (cm)	111 ± 13	110 ± 12	113 ± 13	110 ± 12	0.68	0.58	0.88
Waist: hip ratio	1.0 ± 0.1	1.0 ± 0.1	1.0 ± 0.1	1.0 ± 0.1	1.00	0.82	0.82
**Exercise capacity**							
HR_max_ (beats/min)	169 ± 10	171 ± 10	169 ± 11	169 ± 12	0.88	0.85	0.89
Workload_max_ (watt)	232 ± 52	275 ± 69	197 ± 51	245 ± 69	**0.048**	**0.01**	0.86
*V̇*O_2peak_ (L)	2.9 ± 0.7	3.4 ± 0.7	2.5 ± 0.6	3.1 ± 0.9	0.05	**0.01**	0.76
*V̇*O_2peak_ (mL/min/kg)	27.5 ± 3.6	32.3 ± 3.6	24.0 ± 3.8	30.1 ± 5.7	**0.02**	**< 0.001**	0.62
*V̇*O_2peak_ (mL/min/kg FFM)	44.9 ± 3.6	51.7 ± 3.6	39.1 ± 5.5	47.8 ± 7.3	**< 0.001**	**< 0.001**	0.53
**Strength**							
Leg power (W)	256 ± 113^b^	272 ± 115^b^	207 ± 87^c^	204 ± 69^c^	0.05	0.81	0.74
Leg power (W/kg FFM)	3.7 ± 1.2^b^	3.8 ± 1.1^b^	3.3 ± 1.1^c^	3.2 ± 0.8^c^	0.11	0.64	0.70
Handgrip strength (kg)	42 ± 13^b^	41 ± 13^b^	40 ± 12^b^	38 ± 10^b^	0.51	0.69	0.98
30‐s sit‐to‐stand (reps)	18 ± 3^b^	20 ± 4^b^	17 ± 3^c^	19 ± 3^c^	0.26	**0.03**	0.68
**Plasma biochemical characteristics**							
HbA_1c_ (mmol/mol)	37 ± 4	38 ± 3	62 ± 18	60 ± 21	**< 0.001**	0.93	0.21
TC (mmol/L)	5.1 ± 0.9	4.9 ± 0.7	4.0 ± 0.9	4.0 ± 0.8	**< 0.001**	0.60	0.67
LDL‐C (mmol/L)	3.3 ± 0.8	3.3 ± 0.7	2.4 ± 0.8	2.4 ± 0.8	**< 0.001**	0.98	0.80
HDL‐C (mmol/L)	1.2 ± 0.3	1.3 ± 0.4	1.2 ± 0.4	1.2 ± 0.3	0.25	0.73	0.95
Triglycerides (mmol/L)	1.7 ± 0.9	1.2 ± 0.4	1.9 ± 1.2	1.5 ± 0.6	0.26	0.20	0.69
hsCRP (mg/L)	2.7 ± 1.8	2.9 ± 1.8	2.7 ± 3.1	2.4 ± 2.4	0.21	0.91	0.80
**Medication**							
Glucose‐lowering (no.)	0		13				
Antihypertensive (no.)	1		7				
Cholesterol‐lowering (no.)	2		8				

*Note:* Data are presented as mean ± SD. Leg power and handgrip strength are pooled right and left side results. Details of the medication are given in the Section 2. HbA1c was log‐transformed for statistical analysis; descriptive values are presented on the original scale. Bold values indicate statistically significant *p*‐values.

Abbreviations: CON, control group; FFM, fat‐free mass; HDL‐C, HDL‐cholesterol; HR_max_, maximal heart rate; hsCRP, high‐sensitivity C‐reactive protein; LDL‐C, LDL‐cholesterol; T2D, type 2 diabetes group; TC, total cholesterol; VAT, visceral adipose tissue.

^a^ *T*‐test.

^b^ *n* = 12.

^c^ *n* = 11.

### 3.2 Exercise Capacity

The difference in absolute *V̇*O_2peak_ between the groups was not statistically significant (*p* = 0.05), while *V̇*O_2peak_ relative to body mass and fat‐free mass (FFM) were lower in the diabetes group (*p* = 0.02 and *p* < 0.001, respectively). Both absolute (*p* = 0.01) and relative (*p* < 0.001) *V̇*O_2peak_ increased with training, and so did the maximal workload obtained during the *V̇*O_2peak_ test (*p* = 0.01) (Table 1). As expected, there were no differences between the groups and no effect of training on the maximal heart rate obtained during a *V̇*O_2peak_ test. The *V̇*O_2peak_ progress was monitored with tests every 3 weeks and is presented in Figure S2.

### 3.3 Strength and Functional Performance

There was no effect of group or training on leg power or handgrip (Table 1). However, the number of repetitions during a 30‐s sit‐to‐stand test increased significantly in response to training (*p* = 0.03).

### 3.4 Adherence to Training

The attendance rates in the control and diabetes groups were 99% ± 5% and 98% ± 4%, respectively. Table S4 shows the quality (based on duration and average heart rate) of each training session. The controls trained at higher workloads than the diabetes group (*p* = 0.01), but there was no significant (*p* = 0.05) difference between the groups in increase in average workload during the training period (controls: 21%; diabetes: 31%) (Table S5). There was no effect of group or training on average heart rate or the percentage of heart rate reserve they obtained during the training sessions.

### 3.5 Glucose Metabolism

#### 3.5.1 Glucose Clearance

During steady state at the first clamp step, glucose clearance increased by 17% in controls (from 4.7 ± 2.9 to 5.4 ± 3.0 mL/min/kg FFM) and by 56% in the T2D group (from 2.6 ± 1.0 to 4.0 ± 1.7) (Figure 2a). At the second clamp step, clearance increased by 11% in controls (from 13.8 ± 2.5 to 15.2 ± 2.6) and by 30% in the T2D group (from 10.9 ± 2.9 to 14.2 ± 2.9) (Figure 2a). Repeated‐measures ANOVA revealed significant main effects of group and training at both steps (Figure 2b). Sensitivity analyses using linear mixed‐effects models, which included all available participants, confirmed these findings: training increased clearance in both groups, with larger absolute improvements in T2D (+1.43 [0.88–1.97] at Step 1; +3.18 [2.12–4.23] at Step 2) than in controls (+0.76 [0.21–1.31] at Step 1; +1.49 [0.53–2.45] at Step 2). The between‐group difference in training response reached significance at Step 2 (*p* = 0.021) but not at Step 1 (*p* = 0.093) (Table S3).

**FIGURE 2:**
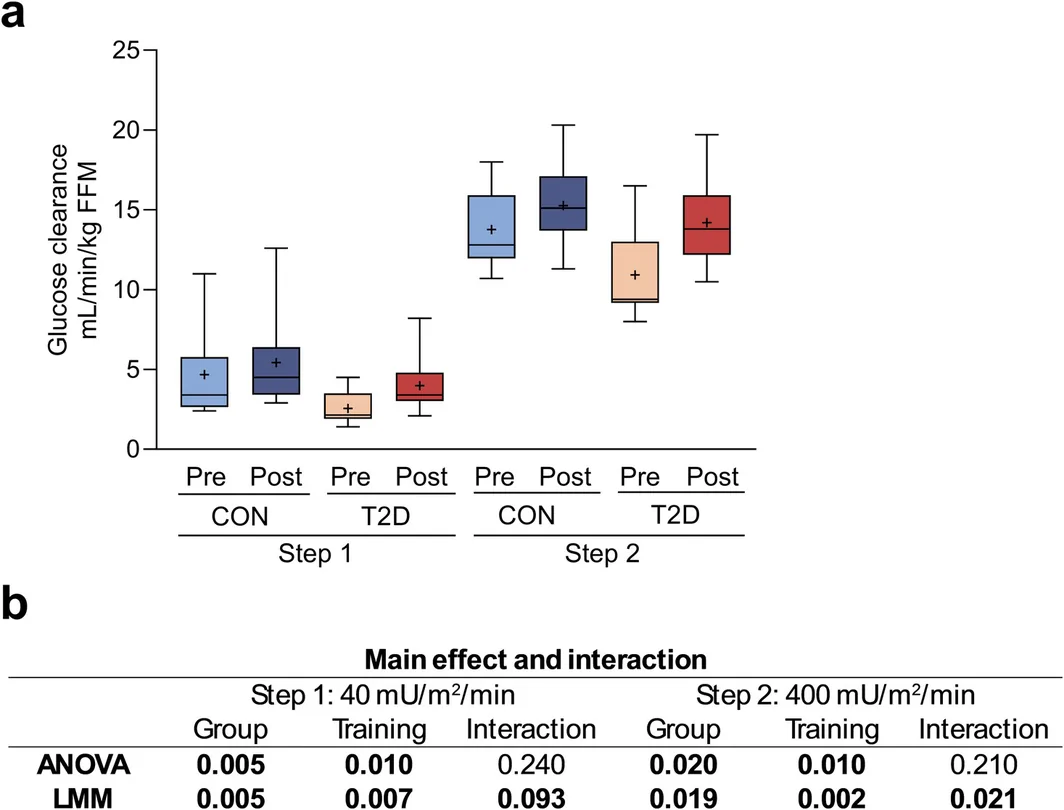
Glucose clearance. (a) Box‐and‐whiskers plot showing glucose clearance before (Pre) and after (Post) 12 weeks of endurance training in individuals with type 2 diabetes (T2D) and in a control group (CON) matched for age and BMI. Glucose clearance was measured during a hyperinsulinaemic isoglycaemic clamp at insulin infusion rates of 40 mU/m^2^/min (Step 1: CON, *n* = 10; T2D, *n* = 10) and 400 mU/m^2^/min (Step 2: CON, *n* = 10; T2D, *n* = 8). The box represents the interquartile range with the median (horizontal line) and mean (+) inside; whiskers indicate the full data range. (b) Group means ± SD at baseline (Pre) and after training (Post), with *p*‐values of the main effects (Group, Training) and the Group × Training interaction shown for each clamp step. Both repeated‐measures analysis of variance (ANOVA) and linear mixed‐effects models (LMMs) are reported. ANOVA showed significant main effects of group and training at both steps, but no significant interaction. LMMs, which included all available participants, confirmed significant training effects in both groups, with greater improvements in T2D at Step 2 (interaction *p* = 0.021). Full model estimates are given in Table S3. ANOVA, analysis of variance; CON, control group;FFM, fat‐free mass; LMM, linear mixed‐effects model; Post, after training; Pre, before training; T2D, type 2 diabetes.

#### 3.5.2 Forearm Blood Flow and Glucose Uptake

We measured basal and insulin‐stimulated forearm blood flow in a subgroup of the participants (*n* = 5 in each group). Blood flow, glucose extraction, and glucose uptake increased in response to insulin stimulation (*p* < 0.001) but with no effect of group or training (Figure S3).

#### 3.5.3 Plasma Glucoregulatory Hormones and Metabolites

There was a group effect on plasma concentrations of glucose (*p* < 0.001), proinsulin (*p* < 0.001), C‐peptide (*p* = 0.003), NEFA (*p* = 0.002), glycerol (*p* < 0.001), and ketone bodies (*p* < 0.001), all of which were higher in the diabetes group, whilst adiponectin was lower (*p* < 0.001) (Figure S4). Proinsulin (*p* = 0.02) and NEFA (*p* = 0.02) concentrations decreased in response to training. The plasma concentrations of growth hormone and insulin increased, while proinsulin, C‐peptide, NEFA, glycerol, and ketone bodies decreased significantly in response to insulin stimulation (*p* < 0.001 for all). Plasma insulin concentrations during the clamp at baseline, Steps 1 and 2 are shown in Figure S4.

### 3.6 Substrate Use and Metabolic Flexibility

#### 3.6.1 During Insulin Stimulation

Resting energy expenditure, RER, glucose, and fat and protein oxidation were similar in the two groups (Table 2). Resting energy expenditure, RER, glucose oxidation, and non‐oxidative glucose disposal increased significantly with insulin stimulation (for all: *p* < 0.001), while fat oxidation decreased significantly with insulin stimulation (*p* < 0.001). A post hoc analysis of non‐oxidative glucose disposal showed significant within‐group differences between the two clamp steps. The control and diabetes groups displayed similar levels of metabolic flexibility, and there was no effect of training (Figure 3a).

**TABLE 2:** Resting energy expenditure and substrate utilization, basal and during a 2‐step hyperinsulinaemic–isoglycaemic clamp (insulin infusion rate 40 mU/m^2^/min [Step 1] and 400 mU/m^2^/min [Step 2]) in a control (CON) and type 2 diabetes (T2D) group.

	Basal		Clamp Step 1		Clamp Step 2		Main effect			Interaction (three‐way)
	Pre	Post	Pre	Post	Pre	Post	Group	Training	Insulin	
**Energy expenditure (kcal/d)**										
CON	1964 ± 480	1931 ± 342	2123 ± 325	2097 ± 289	2389 ± 402	2311 ± 369	0.76	0.97	**< 0.001**	0.99
T2D	2016 ± 351	2105 ± 352	1996 ± 274	2046 ± 268	2368 ± 321	2392 ± 387				
***V̇*O**_**2**_ **(mL/min)**										
CON	284 ± 71	279 ± 50	307 ± 49	303 ± 42	337 ± 60	327 ± 53	0.48	0.90	**< 0.001**	1.00
T2D	284 ± 56	297 ± 59	274 ± 52	284 ± 50	330 ± 48	337 ± 56				
**RER**										
CON	0.84 ± 0.08	0.82 ± 0.07	0.82 ± 0.08	0.83 ± 0.05	0.94 ± 0.06	0.92 ± 0.05	0.80	0.34	**< 0.001**	0.81
T2D	0.82 ± 0.08	0.80 ± 0.05	0.84 ± 0.07	0.82 ± 0.07	0.95 ± 0.12	0.95 ± 0.08				
**Glucose oxidation (mg/min)**										
CON	136 ± 65	119 ± 72	159 ± 94	151 ± 68	328 ± 63	299 ± 82	0.28	0.38	**< 0.001**	0.94
T2D	122 ± 97	114 ± 75	166 ± 102	156 ± 109	347 ± 200	352 ± 156				
**Fat oxidation (mg/min)**										
CON	62 ± 48	65 ± 30	62 ± 41	61 ± 23	22 ± 37	27 ± 28	0.12	0.72	**< 0.001**	0.18
T2D	67 ± 40	77 ± 37	49 ± 41	60 ± 48	0 ± 81	2 ± 49				
**Protein oxidation (mg/min)**										
CON	51 ± 24	57 ± 28	52 ± 15	56 ± 13	50 ± 19	50 ± 24	0.65	0.79	0.37	0.97
T2D	48 ± 22	49 ± 20	50 ± 17	49 ± 12	75 ± 50	71 ± 35				
**Non‐oxidative glucose disposal (mg/min)**										
CON	—	—	269 ± 227	274 ± 279	708 ± 191^†††^	795 ± 182^†††^	0.35	0.31	**< 0.001**	**< 0.001**
T2D	—	—	65 ± 120*	162 ± 126	864 ± 312^†††^	1001 ± 232^†††^				

*Note:* Sample sizes: Basal: *n* = 14 (CON) and *n* = 13 (T2D), Step 1: *n* = 11 (CON) and *n* = 7 (T2D), Step 2: *n* = 10 (CON) and *n* = 7 (T2D). Significance annotations for post hoc analysis in case of significant interaction: CON versus T2D: *< 0.05. Within group Steps 1 versus 2: ^†††^< 0.001. Bold values indicate statistically significant *p*‐values.

**FIGURE 3:**
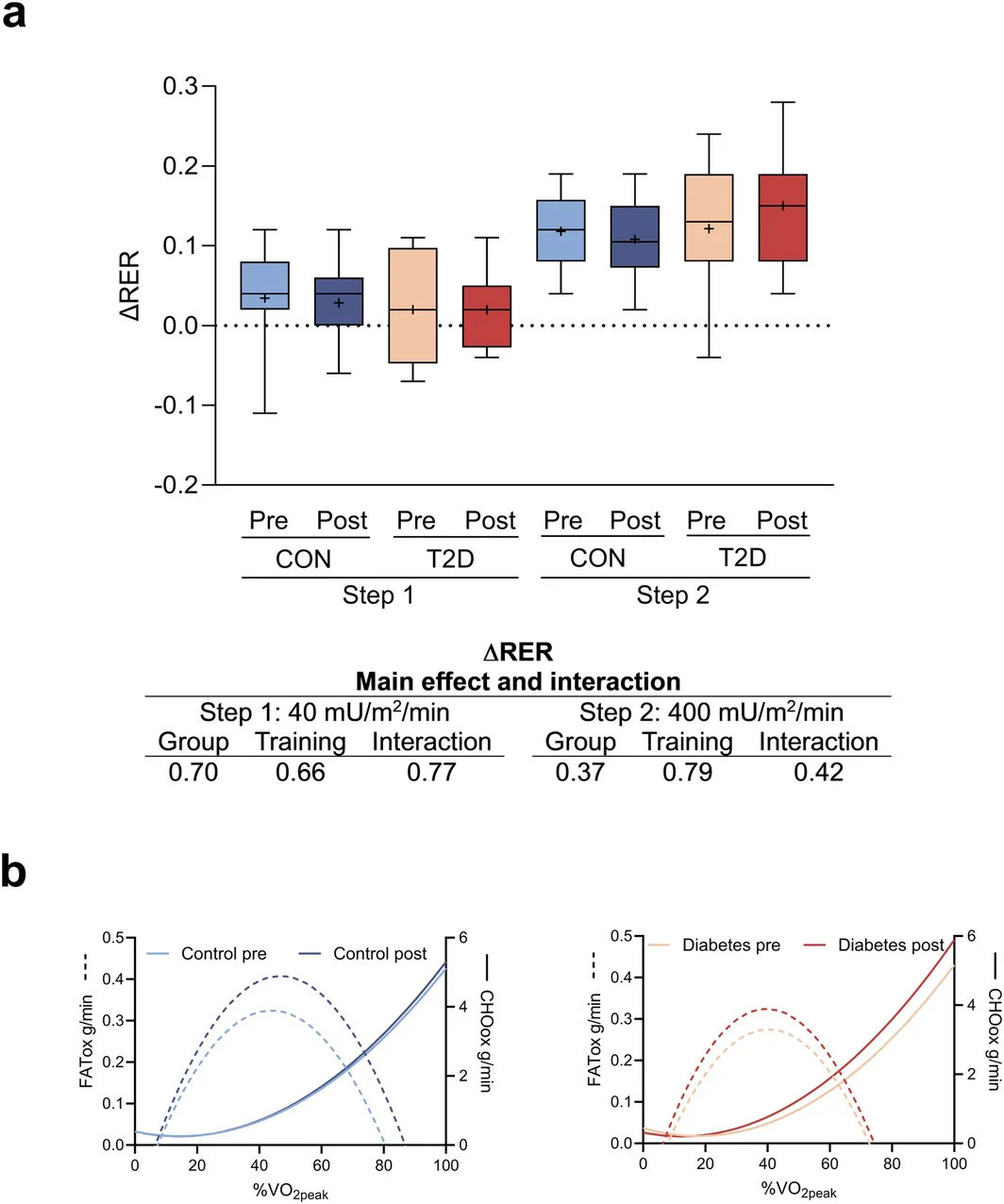
Metabolic flexibility during (a) insulin stimulation and (b) exercise. (a) The change in respiratory exchange ratio (ΔRER) from basal to insulin stimulation at 40 mU/m^2^/min (CON: *N* = 7, T2D: *N* = 8) and 400 mU/m^2^/min (CON: *N* = 10, T2D: *N* = 7). The box represents the interquartile range with the median (horizontal line) and average (+) within. The whiskers encompass the full range of the data. (b) Average fat and carbohydrate (CHO) oxidation rates measured during an incremental bicycle ergometer test (CON: *N* = 14, T2D: *N* = 15).

#### 3.6.2 During Exercise

Metabolic flexibility during exercise is illustrated in Figure 3b. PFO was generally lower in the diabetes group compared with the control group (*p* = 0.03, main effect) and increased in response to training by ~26% and ~17% in the control and the diabetes group, respectively (*p* = 0.01). Before and after training PFO was 0.33 ± 0.09 and 0.42 ± 0.08 in the control group and 0.30 ± 0.07 and 0.35 ± 0.12 g/min in the diabetes group, respectively, with no interaction between group and time (*p* = 0.42). The intensity at which PFO occurred was similar in the two groups (*p* = 0.10) with no effect of training (*p* = 0.28) (control: 43% ± 5% to 48% ± 7% of *V̇*O_2peak_; diabetes: 41% ± 9% to 42% ± 11% of *V̇*O_2peak_).

### 3.7 Skeletal Muscle Mitochondrial Content, Respiratory Capacity, and H_2_O_2_ Emission Capacity

CS activity was higher in the control group at baseline (*p* = 0.01) and increased in response to training in both groups (*p* < 0.001) (Figure 4a). HAD activity was higher in the control group at baseline (*p* = 0.02) and increased in response to training only in the diabetes group (*p* = 0.04). Training increased mass‐specific (*p* < 0.001) (Figure 4b) and decreased mitochondria‐specific respiratory capacity (*p* < 0.001) (Figure 4c). There were no group differences in either measure or in mitochondria‐specific or respiration‐specific H_2_O_2_ emission, both of which decreased with training (*p* < 0.001) (Figure 4d,e).

**FIGURE 4:**
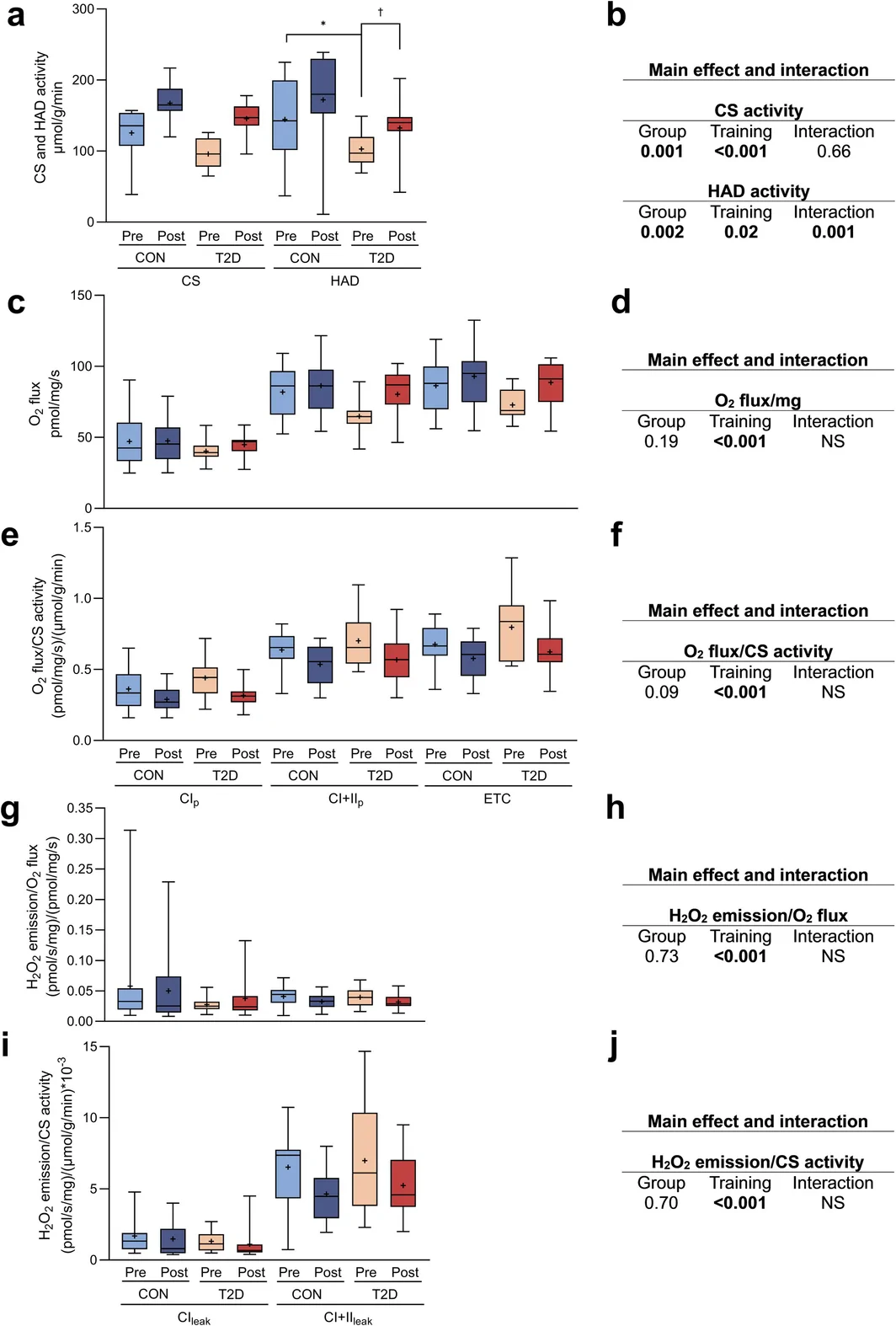
Mitochondrial function (O_2_ flux and H_2_O_2_ emission) in skeletal muscle of individuals with type 2 diabetes (T2D) and a control group (CON) before (pre) and after (post) training. (a) CS and HAD activity (CON: *n* = 12, T2D: *n* = 15). (b) Corresponding statistical results for CS and HAD activity. (c) O_2_ flux normalized to mg of tissue (CON: *n* = 13, T2D: *n* = 15). (d) Corresponding statistical results for O_2_ flux normalized to tissue mass. (e) O_2_ flux normalized to CS activity (CON: *n* = 10, T2D: *n* = 15). (f) Corresponding statistical results for O_2_ flux normalized to CS activity. (g) H_2_O_2_ (ROS) emission normalized to O_2_ flux (CON: *n* = 14, T2D: *n* = 14). (h) Corresponding statistical results for H_2_O_2_ emission normalized to O_2_ flux. (i) H_2_O_2_ (ROS) emission normalized to CS activity (CON: *n* = 11, T2D: *n* = 14). (j) Corresponding statistical results for H_2_O_2_ emission normalized to CS activity. Panels (b), (d), (f), (h), and (j) present the *p*‐values for the main effects of group and training and their interaction. The boxes represent the interquartile range, with the median (horizontal line) and mean (+) indicated. The whiskers encompass the full range of the data. For panels (c) and (e), the statistical analysis also included leak respiration (CIleak), in addition to the respiratory states shown. For panels (g) and (i), the statistical analysis additionally included two steps with lower succinate concentrations (1 and 2 mmol/L; CI+IIleak), which are not shown for clarity. Panels (c) and (e) share the same x‐axis, as do panels (g) and (i). Significance annotations for post hoc comparisons in the case of significant interactions: CON versus T2D: *p < 0.05; within‐group pre versus post: ^†^ *p* < 0.05. For panels (c), (e), (g), and (i), the main effect of respiratory state was significant (all *p* < 0.001). CI, mitochondrial complex I; CI+II, mitochondrial complexes I and II; CS, citrate synthase; ETC, electron transport chain; HAD, hydroxyacyl‐CoA dehydrogenase; NS, non‐significant; ROS, reactive oxygen species.

## 4 Discussion

The principal findings of this study were that insulin sensitivity and metabolic flexibility to fat, but not glucose, were lower in the diabetes group, but increased in both groups with training. Consistent with our hypotheses, mitochondrial respiratory capacity and H_2_O_2_ emission were similar in individuals with and without T2D. Interestingly, the absolute (mass‐specific) respiration increased while the relative (mitochondria‐specific) respiration and H_2_O_2_ emission decreased in both groups with training.

### 4.1 Insulin Sensitivity

In agreement with previous studies [18, 19, 25], glucose clearance was lower in the diabetes group, and higher after than before training. However, because no glucose tracer was used, endogenous glucose production could not be quantified separately from peripheral glucose disposal. This is particularly relevant during Step 1, where incomplete suppression of endogenous glucose production may contribute to the calculated glucose clearance, especially in T2D. Glucose delivery may have a role in insulin sensitivity, but resting and insulin‐stimulated forearm blood flow and glucose extraction were similar in the two groups and remained unaltered by training. Thus, glucose delivery likely did not explain the observed differences in glucose clearance.

### 4.2 Metabolic Flexibility

T2D has been associated with impaired metabolic flexibility [11, 12] but the changes in RER during the hyperinsulinaemic, isoglycemic clamp were similar in the groups at both physiological and supraphysiological insulin levels. This is in opposition to some studies [1, 19, 26]. A key methodological difference is that we performed clamps under isoglycaemic conditions, which more closely reflect the typical metabolic state of individuals with T2D than strict euglycaemia. Additionally, extra insulin boluses or a longer clamp duration for the diabetes group were not required to reach euglycaemia. Our results are consistent with those of Vind et al., who compared euglycaemic and isoglycaemic clamps and found that impaired metabolic flexibility in T2D was apparent during euglycaemia but not during isoglycaemia [7]. Correcting for glucose disposal provides a more appropriate estimate of tissue glucose availability and has been shown to eliminate the impaired metabolic flexibility observed in individuals with T2D compared to a proper control group [17, 27, 28].

Moreover, correcting measures of metabolic flexibility for whole‐body glucose disposal provides a better estimate of tissue glucose availability. Studies that account for glucose disposal have reported that the apparent impairment in metabolic flexibility in people with T2D can be eliminated when an appropriate control group and correction are applied [27, 28]. Taken together, these methodological differences—clamp glycaemic target and adjustment for glucose disposal—likely explain at least part of the discrepancy between our findings and some earlier reports.

In line with prior training studies in both healthy subjects [29] and patients with T2D [18] metabolic flexibility with respect to glucose (i.e., the insulin‐induced change in RER) did not change after the exercise intervention in our cohort. Some previous studies have reported increases in delta RER with training [1, 19]; however, in at least one of these reports the change was driven primarily by a lower basal RER rather than an augmented insulin response, implying increased basal fat oxidation. Basal RER and substrate use can be influenced by recent dietary carbohydrate intake [30], and diet was not fully controlled or reported in some earlier studies [19], which could contribute to inconsistent findings. More recently, Garthwaite et al. [31] reported improved insulin‐stimulated metabolic flexibility in individuals with metabolic syndrome who successfully reduced their sedentary time by at least 30 min/day, although no significant change in ΔRER was observed in the randomized intervention group as a whole. Together, these findings indicate that metabolic flexibility may respond not only to structured exercise but also to changes in habitual physical activity and sedentary behavior.

Galgani & Fernández‐Verdejo suggested that there should be more focus on metabolic flexibility to fat instead of glucose [5]. Peak fat oxidation (PFO) during exercise may provide an exercise‐based surrogate of metabolic flexibility toward lipid utilization, although it reflects maximal fat‐oxidation capacity rather than substrate switching per se. In our study, PFO was lower in the diabetes group at baseline, indicating a reduced capacity for fat oxidation during exercise. Previous reports on PFO in T2D are mixed, with some studies finding lower PFO [15, 32] and others reporting no difference from controls [14, 16]. Differences in dietary composition [33] and cardiorespiratory fitness (*V̇*O_2peak_) [34] may explain some of the discrepancies.

Notably, training increased PFO in the diabetes group (a finding supported by the increase in HAD activity only in T2D), consistent with two previous studies [14, 35]. Although T2D has traditionally been associated with impaired metabolic flexibility, a recent systematic review and meta‐analysis suggests that the impairment is more strongly related to obesity than to the T2D diagnosis itself [17]. Nevertheless, our results indicate a substrate‐specific component: flexibility assessed by insulin‐stimulated glucose oxidation (clamp RER) was similar between groups under isoglycaemic conditions, whereas flexibility assessed by fat oxidation (PFO) was reduced in T2D and responsive to training. In other words, PFO paralleled group differences in glucose clearance and the training‐induced increases observed in both groups, supporting the idea that fat oxidation measures may be a more sensitive index of metabolic flexibility in T2D than clamp‐derived RER alone.

### 4.3 Mitochondrial Function

Impaired mitochondrial respiratory capacity has been implicated in insulin resistance [2] despite studies questioning this relationship [6, 8, 9, 10]. We could not detect a difference in mass‐specific respiration between the diabetes and control groups. However, a lower CS activity, a marker of mitochondrial content, was observed in the diabetes group. This has been shown before [2, 24], and here, we suspect that the differences in *V̇*O_2peak_ are the underlying reason, as several studies with groups well‐matched for *V̇*O_2peak_ found no differences in CS activity [4, 8, 10, 14, 25]. After training, CS activity and mass‐specific respiratory capacity increased as previously observed [3, 8, 14, 36].

The groups in our study had similar mitochondria‐specific respiration (normalized to CS activity), suggesting that this mitochondrial function is not impaired by T2D per se. Interestingly, both groups showed a decrease in mitochondria‐specific respiration after training. Such a decrease has been reported in healthy individuals following 6 weeks of endurance training [37]. A higher mitochondria‐specific respiratory capacity and higher ROS production have been shown in insulin‐resistant individuals with obesity compared to lean insulin‐sensitive controls [38], and an increase in mitochondria‐specific respiration was observed in young, healthy males after 4 days of bed rest [39]. These findings imply that high mitochondria‐specific respiration can be an expression of worsened metabolic health and stressed mitochondria. Conversely, a lower workload for each mitochondrion may result in healthier and less stressed mitochondria. This hypothesis is supported by the lower mitochondria‐specific H_2_O_2_ emission observed after training in the present study. However, this interpretation should be made cautiously, as mitochondria‐specific values were normalized to citrate synthase activity, which is only an indirect marker of mitochondrial content. Additionally, H_2_O_2_ emission normalized to respiration decreased, suggesting a more efficient electron transport chain. Mass‐specific H_2_O_2_ increased after training, probably as an expression of the overall increased respiration.

Although mitochondria are central to substrate utilization, our results show no simple, consistent relationship between mitochondrial O_2_ flux and metabolic flexibility. Specifically, glucose‐related metabolic flexibility (clamp) did not differ between T2D and controls and was not altered by training. In contrast, fat‐related metabolic flexibility (PFO with exercise) was lower in T2D than controls and improved with training. Mitochondrial mass‐specific O_2_ flux was similar in T2D and controls but increased significantly after training. Thus, responses differ by substrate: glucose‐related flexibility shows no group or training effects, whereas fat‐related flexibility does. These divergent findings indicate that a single, uniform pattern linking mitochondrial O_2_ flux to metabolic flexibility across substrates and groups cannot be inferred from our results.

Consistent with this interpretation, adjustment for glucose disposal has been shown to abolish the relationship between mitochondrial function and metabolic flexibility to glucose, implying that substrate availability rather than intrinsic mitochondrial capacity governs substrate switching during insulin stimulation [28]. Conversely, other data show that mitochondrial function determines basal RER [28], suggesting a more prominent role of mitochondria in substrate use during fasting conditions than during insulin stimulation.

### 4.4 Strengths and Weaknesses

A key strength of this study was the combined use of the hyperinsulinaemic clamp and high‐resolution respirometry and the examination of two different types of metabolic flexibility. Although high‐resolution respirometry has the disadvantage of being performed ex vivo, it allowed us to examine the electron transport chain in detail in a tightly controlled environment. A limitation of this study was that, although the group characteristics were similar, exercise capacity (*V̇*O_2peak_) was lower in the diabetes group. Additionally, the diabetes group had a different medication profile, which may have affected the results. The findings are likely generalizable to middle‐aged, sedentary adults with T2D and similar BMI, but may not apply to younger, leaner, or more physically active populations. Although the study was adequately powered for the primary endpoint, it was not powered to detect small between‐group differences in secondary mitochondrial outcomes. Furthermore, citrate synthase activity was used as a surrogate marker of mitochondrial content, although it does not perfectly reflect mitochondrial number or ultrastructural characteristics.

## 5 Conclusion

In summary, our findings indicate that type 2 diabetes is associated with reduced metabolic flexibility for fat oxidation rather than for glucose. Mitochondrial respiratory capacity per se was not impaired in individuals with T2D. However, we report for the first time a training‐induced decrease in mitochondria‐specific respiration in people with T2D, accompanied by reduced H_2_O_2_ emission relative to citrate synthase activity and mitochondrial respiration, which together suggest improved mitochondrial efficiency or health after training. Future studies should ensure careful matching of groups for *V̇*O_2peak_.

### 5.1 Perspective

Future studies should further disentangle the effects of type 2 diabetes, adiposity, and cardiorespiratory fitness on metabolic flexibility and mitochondrial function by matching groups more closely for *V̇*O_2_peak and by incorporating direct measures of mitochondrial content and ultrastructure alongside enzyme‐based surrogate markers. Such approaches may help distinguish effects related to diabetes per se from those attributable to differences in fitness or mitochondrial abundance.

A key research question is whether metabolic flexibility should be considered a substrate‐ and context‐specific phenotype rather than a single characteristic. Direct comparisons of isoglycaemic and euglycaemic clamps with measures of fat oxidation during exercise, ideally combined with standardized dietary control, may clarify which components of metabolic flexibility are most closely related to insulin resistance and training responsiveness.

The physiological significance of changes in mitochondria‐specific respiration and ROS production also warrants further investigation. Combining high‐resolution respirometry with measures of mitochondrial density, coupling efficiency, redox regulation, and ultrastructure may help determine whether lower respiration per unit of mitochondrial content reflects improved mitochondrial efficiency, reduced mitochondrial stress, or other adaptations to endurance training.

## Funding

This work was supported by Novo Nordisk.

## Ethics Statement

All participants gave written informed consent before participation, and the study was performed according to the guidelines of the Declaration of Helsinki, approved by the Regional Scientific Ethical Committees for the Capital Region of Denmark (H‐20046605), and registered at ClinicalTrials.gov (NCT04945551).

## Conflicts of Interest

The authors declared the following potential conflicts of interest with respect to research, authorship, and/or publication of this article: Thorbjörn Åkerström is a paid employee at Novo Nordisk A/S. Novo Nordisk A/S covered salaries and running expenses, which were administered by the University of Copenhagen and had no influence on execution, analysis, or interpretation of the work. The authors declare no conflicts of interest.

## Supporting information


**Table S1:** TREND checklist.
**Table S2:** Post hoc power analysis.
**Table S3:** Linear mixed‐effects models.
**Table S4:** All training sessions for each participant.
**Table S5:** Heart rate and workload.
**Figure S1:** TREND Flow diagram of enrollment.
**Figure S2:** sms70383‐sup‐0001‐Supinfo.docx. *V̇*O_2peak_.
**Figure S3:** Forearm A‐V balance study.
**Figure S4:** Hormones, substrates and metabolites during clamp.

## Data Availability

Data sets generated during the current study are available from the corresponding author upon reasonable request.
