# Supplementary material for: Endurance Training Improves Insulin Sensitivity, Metabolic Flexibility, and Mitochondrial Function in Type 2 Diabetes
Source: Scand J Med Sci Sports. 2026 Oct 24;36(11):e70383. doi: 10.1111/sms.70383 (PMC13695046; doi:10.1111/sms.70383)
Supplement: Supplementary file 1 [file SMS-36-e70383-s001.docx]

# Supplementary Material – Tables and Figures

Table S1:	TREND checklist.

Table S2:	Post hoc power analysis

Table S3:	Linear mixed-effects models.

Table S4:	All training sessions for each participant.

Table S5:	Heart rate and workload.

Fig S1:	TREND Flow diagram of enrollment

Fig S2:	V̇O_2peak_

Fig S3:	Forearm A-V balance study.

Fig S4:	Hormones, substrates and metabolites during clamp.

# Table S1

# TREND Checklist

| Item No. | TREND Checklist Item | Pg # | Notes |
| --- | --- | --- | --- |
| 1 | Title and Abstract – Information on how units were allocated to interventions | pp. 1-3 | Abstract includes design |
| 2 | Background – Scientific background and rationale | pp. 4-5 | Introduction provides rationale for insulin sensitivity, metabolic flexibility, mitochondria |
| 3 | Participants – Eligibility criteria, recruitment methods | p. 5 | Methods: Study population |
| 4 | Interventions – Details of interventions intended for each group | p. 6 | Methods: Exercise intervention |
| 5 | Objectives – Specific objectives and hypotheses | pp. 4-5 | Introduction, final paragraph |
| 6 | Outcomes – Clearly defined primary and secondary outcomes | pp. 10-11 | Methods: Outcomes; Statistical analyses |
| 7 | Sample size – How sample size was determined | pp. 10-11 | Methods: Statistical analyses; post hoc power analysis |
| 8 | Assignment method – Unit of assignment, method used | p. 5 | Nonrandomized; groups defined by diabetes status |
| 9 | Blinding – Whether participants, administrators, outcome assessors blinded | p. 10 | Methods: Statistical analyses, blinding description |
| 10 | Unit of analysis – How data were analyzed (individual/group level) | p. 10-11 | Individual level; LMM models |
| 11 | Statistical methods – Statistical methods used to compare groups | pp. 10-11 | ANOVA, LMM, transformations, post hoc tests |
| 12 | Participant flow – Numbers of participants completing each stage | p. 5 + Fig. S1 | Results: participant flow described; TREND Flow diagram shown. |
| 13 | Recruitment – Dates defining recruitment and follow-up | p. 5 | Methods: Experimental protocol (2021–2023) |
| 14 | Baseline data – Baseline demographic and clinical characteristics | p. 11 + Table 1 | Results: Table 1 |
| 15 | Numbers analyzed – Number of participants in each analysis | p.11+ | Results.Legends to figures and tables; Supporting Information Table S2 |
| 16 | Outcomes and estimation – Results for each primary/secondary outcome | pp. 11–13 | Results main text |
| 17 | Ancillary analyses – Subgroup/sensitivity analyses | pp. 10–11 | Results: Glucose clearance; Supplementary Table S2 |
| 18 | Adverse events – Important adverse events or side effects | p. 11 | Results: safety statement (no serious adverse events) |
| 19 | Interpretation – Interpretation of results, considering hypotheses and bias | pp. 13–17 | Discussion |
| 20 | External validity – Generalizability of findings | p. 17 | Perspectives |
| 21 | Overall evidence – How results fit with current evidence | pp. 13–17 | Discussion, Research in context |
| 22 | Funding – Sources of support, role of funders | p. 1 | Funding statement. |

## Supplementary Table S2. Post hoc power analysis for the primary outcome (glucose clearance)

### Within-group (paired)

| Clamp step | Group | n | Mean PRE | Mean POST | Mean change | Pre–post r | Cohen dz | Power (exact) |
| --- | --- | --- | --- | --- | --- | --- | --- | --- |
| Step 1 | T2D | 10 | 2.55 ± 0.98 | 3.99 ± 1.74 | 1.44 ± 0.99 | 0.88 | 1.45 | 0.98 |
| Step 1 | Control | 10 | 4.67 ± 2.90 | 5.44 ± 3.04 | 0.77 ± 0.90 | 0.96 | 0.86 | 0.68 |
| Step 2 | T2D | 8 | 10.93 ± 2.86 | 14.20 ± 2.91 | 3.27 ± 1.18 | 0.92 | 2.77 | 1.00 |
| Step 2 | Control | 10 | 13.77 ± 2.49 | 15.26 ± 2.56 | 1.49 ± 1.95 | 0.70 | 0.76 | 0.58 |

### Between-group (change scores)

| Clamp step | n  T2D | n  Control | Mean Δ T2D | Mean Δ Control | Hedges g | Power (exact) |
| --- | --- | --- | --- | --- | --- | --- |
| Step 1 | 10 | 10 | 1.44 ± 0.99 | 0.77 ± 0.90 | 0.68 | 0.32 |
| Step 2 | 8 | 10 | 3.27 ± 1.18 | 1.49 ± 1.95 | 1.03 | 0.57 |

Exact noncentral-t power analyses for glucose clearance during clamp Step 1 and Step 2. Within-group (paired) analyses use only complete pre–post pairs. Between-group analyses compare change scores. Effect sizes are reported as Cohen’s dz (paired) and Hedges’ g (between-group). Values are mean ± SD. Units: mL·min⁻¹·kg FFM⁻¹.

## Supplementary Table S3. Linear mixed-effects models (all available participants)

## Step 1

| **Parameter** | **Estimate (95% CI)** | **p-value** | **Interpretation** |
| --- | --- | --- | --- |
| Intercept | 4.83 (3.71 to 5.96) | <0.001 |  |
| Group[T.T2D] | -2.15 (-3.65 to -0.65) | 0.005 | Baseline difference (T2D − CON) |
| Time[T.Post] | 0.76 (0.21 to 1.31) | 0.007 | Training effect in CON |
| Group[T.T2D]:Time[T.Post] | 0.67 (-0.11 to 1.44) | 0.093 | Additional improvement in T2D relative to CON |
| Derived: Training effect in T2D | 1.43 (0.88 to 1.97) | — | CON training effect + interaction term (Group[T.T2D]:Time[T.Post]) |
| Group Var | 8.73 | — | Random intercept variance |

### Step 2

| **Parameter** | **Estimate (95% CI)** | **p-value** | **Interpretation** |
| --- | --- | --- | --- |
| Intercept | 13.77 (12.27 to 15.25) | <0.001 |  |
| Group[T.T2D] | -2.37 (-4.34 to -0.40) | 0.019 | Baseline difference (T2D − CON) |
| Time[T.Post] | 1.49 (0.53 to 2.45) | 0.002 | Training effect in CON |
| Group[T.T2D]:Time[T.Post] | 1.69 (0.26 to 3.11) | 0.021 | Additional improvement in T2D relative to CON |
| Derived: Training effect in T2D | 3.18 (2.12 to 4.23) | — | CON training effect + interaction term (Group[T.T2D]:Time[T.Post]) |
| Group Var | 3.77 | — | Random intercept variance |

Linear mixed-effects models (random intercept for subject) were fitted separately for Step 1 and Step 2: Clearance ~ Group × Time. Reference levels: Group=Control, Time=Pre. Estimates are shown as Estimate (95% CI) with p-values. Confidence intervals and p-values are not reported for random-effect variance components (“Group Var”) because variance is constrained to be ≥0, and standard Wald tests are invalid for boundary parameters. Units: mL·min⁻¹·kg FFM⁻¹.

Parameters shown:

- Intercept: Mean value in the reference group (Control at Pre).
- Group[T.T2D]: Difference at baseline (Pre) between the T2D group and the Control group.
- Time[T.Post]: Change from Pre to Post in the Control group (training effect in controls).
- Group[T.T2D]:Time[T.Post]: Additional training effect in the T2D group relative to the Control group (i.e., the group × time interaction).
- Group Var: refers to the estimated variance of the random intercept for subject ID in the linear mixed-effects model (LMM), representing between-participant variability in glucose clearance not explained by the fixed effects.

| Week | 1 | | | 2 | | | 3 | | | 4 | | | 5 | | | 6 | | | 7 | | | 8 | | | 9 | | | 10 | | | 11 | | | 12 | | | 13 | | |  |
| --- | --- | --- | --- | --- | --- | --- | --- | --- | --- | --- | --- | --- | --- | --- | --- | --- | --- | --- | --- | --- | --- | --- | --- | --- | --- | --- | --- | --- | --- | --- | --- | --- | --- | --- | --- | --- | --- | --- | --- | --- |
| Training no. | 1 | 2 | 3 | 4 | 5 | 6 | 7 | 8 | 9 | 10 | 11 | 12 | 13 | 14 | 15 | 16 | 17 | 18 | 19 | 20 | 21 | 22 | 23 | 24 | 25 | 26 | 27 | 28 | 29 | 30 | 31 | 32 | 33 | 34 | 35 | 36 | 37 | 38 | 39 | Attnd.(%) |
| CON1 | ● | ● | ● | ● | ● | ● | ● | ● | ● | ● | ● | ● | ● | ○ | ● | ● | ● | ● | ● | ● | ● | ● | ● | ● | ● | ● | ● | ● | ● | ● | ● | ● | ● | **T** | ● | ● |  |  |  | 97 |
| CON2 | ● | ● | ● | ● | ● | ● | ● | ● | ● | ● | ● | ● | ● | ● | ● | ● | ● | ● | ● | ● | ● | ● | ● | ● | ● | ● | ● | ● | ● | ● | ● | ● | ● | ● | ● | **T** | ● |  |  | 103 |
| CON3 | ● | ● | ● | ● | ● | ● | ● | ● | ● | ● | ● | ● | ● | ● | ● | ● | ● | ● | ● | ● | ◑ | ● | ● | ● | ● | ● | ● | ● | ● | ● | ● | ● | ● | **T** | ○ | ● |  |  |  | 97 |
| CON4 | ● | ● | ● | ● | ● | ● | ● | ● | ● | ○ | ● | ● | ● | ● | ● | ● | ● | ● | ● | ● | ● | ○ | ● | ○ | ● | ● | ● | ○ | ○ | ○ | ○ | ● | ● | ● | **T** | ● |  |  |  | 83 |
| CON5 | ● | ● | ● | ● | ● | ● | ● | ● | ● | ● | ● | ● | ● | ● | ● | ● | ● | ● | ● | ● | ● | ● | ● | ● | ● | ● | ● | ● | ● | ● | ● | ● | ● | **T** | ● | ● |  |  |  | 100 |
| CON7 | ● | ● | ● | ● | ● | ● | ○ | ● | ● | ● | ● | ● | ● | ● | ● | ● | ● | ● | ● | ● | ● | ● | ● | ● | ● | ● | ● | ● | ● | ● | ● | ● | ● | **T** | ● | ● | ● |  |  | 100 |
| CON8 | ● | ● | ● | ● | ● | ● | ● | ● | ● | ● | ● | ● | ● | ● | ● | ● | ● | ● | ● | ● | ● | ● | ● | ● | ● | ● | ● | ● | ● | ● | ● | ● | ● | **T** | ● | ● |  |  |  | 100 |
| CON9 | ● | ● | ● | ● | ● | ● | ● | ● | ● | ● | ● | ● | ● | ● | ● | ● | ● | ● | ● | ● | ● | ● | ● | ● | ● | ● | ● | ● | ● | ● | ● | ● | ● | **T** | ● | ● |  |  |  | 100 |
| CON10 | ● | ● | ● | ● | ● | ● | ● | ● | ◔ | ● | ● | ● | ● | ● | ● | ● | ● | ● | ● | ● | ● | ● | ● | ● | ● | ● | ● | ● | ● | ● | ● | ● | ● | **T** | ● | ● |  |  |  | 100 |
| CON11 | ● | ● | ● | ● | ● | ● | ● | ● | ● | ● | ● | ● | ● | ● | ● | ● | ● | ● | ● | ● | ● | ● | ● | ● | ● | ● | ● | ● | ● | ● | ● | ● | ○ | **T** | ● | ● |  |  |  | 97 |
| CON12 | ● | ● | ● | ● | ● | ● | ● | ● | ● | ● | ● | ● | ● | ● | ● | ● | ● | ● | ● | ● | ● | ● | ● | ● | ● | ● | ● | ● | ● | ● | ● | ● | ● | **T** | ● | ● |  |  |  | 100 |
| CON13 | ● | ● | ● | ● | ● | ● | ● | ● | ● | ● | ● | ● | ● | ● | ● | ● | ● | ● | ● | ● | ● | ● | ● | ● | ● | ● | ● | ● | ● | ● | ● | ● | ● | **T** | ● | ● |  |  |  | 100 |
| CON14 | ● | ● | ● | ● | ● | ● | ● | ● | ● | ● | ● | ● | ● | ● | ● | ● | ● | ● | ● | ● | ● | ● | ● | ● | ● | ● | ● | ● | ● | ● | ● | ● | ● | **T** | ● | ● | ● | ● | ● | 108 |
| CON15 | ● | ● | ● | ● | ● | ● | ● | ● | ● | ● | ◕ | ● | ● | ● | ● | ● | ● | ● | ● | ● | ● | ● | ● | ◕ | ◔ | ● | ● | ● | ● | ● | ● | ● | ◑ | **T** | ● | ● |  |  |  | 100 |
| T2D1 | ● | ● | ● | ● | ● | ● | ● | ● | ● | ● | ● | ● | ● | ● | ● | ● | ● | ● | ● | ● | ● | ● | ● | ● | ● | ● | ● | ● | ● | ● | ● | ● | ● | ● | **T** | ● |  |  |  | 100 |
| T2D2 | ● | ● | ● | ● | ● | ● | ● | ● | ● | ● | ● | ● | ● | ● | ● | ● | ● | ● | ● | ● | ● | ● | ● | ● | ● | ● | ● | ● | ● | ● | ● | ● | ○ | **T** | ● | ● |  |  |  | 97 |
| T2D3 | ● | ● | ● | ● | ● | ● | ● | ● | ● | ● | ● | ● | ● | ● | ● | ● | ● | ● | ● | ◕ | ● | ● | ● | ● | ● | ● | ● | ● | ● | ● | ● | ● | ● | **T** | ● | ● |  |  |  | 100 |
| T2D4 | ● | ● | ● | ● | ● | ● | ● | ● | ● | ● | ● | ● | ● | ● | ● | ● | ● | ● | ● | ● | ● | ● | ● | ● | ● | ● | ● | ● | ● | ● | ● | ● | ● | **T** | ● | ○ |  |  |  | 97 |
| T2D5 | ● | ● | ● | ● | ● | ● | ● | ● | ● | ● | ● | ● | ● | ● | ● | ● | ● | ● | ● | ● | ● | ● | ● | ● | ● | ● | ● | ● | ● | ● | ● | ● | ● | **T** | ○ | ● |  |  |  | 97 |
| T2D6 | ● | ● | ● | ● | ● | ● | ● | ● | ● | ● | ● | ● | ● | ● | ● | ● | ● | ● | ● | ● | ● | ● | ● | ● | ● | ● | ● | ● | ● | ● | ● | ● | ● | ● | **T** | ● |  |  |  | 100 |
| T2D7 | ● | ● | ● | ● | ● | ● | ● | ● | ● | ● | ● | ● | ● | ● | ● | ● | ● | ● | ● | ● | ● | ● | ● | ● | ● | ● | ● | ● | ● | ● | ● | ● | ● | **T** | ● | ● |  |  |  | 100 |
| T2D9 | ● | ● | ● | ● | ● | ● | ● | ● | ● | ● | ● | ● | ● | ● | ● | ● | ● | ◑ | ● | ● | ● | ● | ● | ● | ● | ● | ● | ◑ | ● | ● | ● | ● | ◑ | **T** | ● | ● |  |  |  | 100 |
| T2D10 | ● | ● | ● | ● | ● | ● | ● | ◑ | ● | ● | ● | ● | ● | ● | ● | ● | ● | ● | ● | ● | ● | ● | ● | ● | ● | ● | ● | ● | ● | ● | ● | ● | ● | **T** | ● | ● |  |  |  | 100 |
| T2D13 | ● | ● | ● | ● | ● | ● | ● | ● | ● | ● | ● | ● | ● | ◑ | ● | ● | ● | ● | ● | ● | ● | ● | ● | ● | ● | ● | ◑ | ● | ● | ● | ● | ◕ | ● | **T** | ● | ● |  |  |  | 100 |
| T2D14 | ● | ● | ● | ● | ● | ● | ● | ● | ● | ● | ● | ● | ● | ● | ● | ● | ● | ● | ● | ● | ● | ● | ● | ● | ● | ● | ● | ● | ● | ● | ● | ○ | ● | **T** | ● | ● |  |  |  | 97 |
| T2D15 | ● | ● | ● | ● | ● | ● | ● | ● | ● | ● | ● | ● | ● | ● | ● | ● | ● | ● | ● | ● | ● | ● | ○ | ● | ● | ● | ● | ● | ● | ● | ● | ● | ● | **T** | ● | ● |  |  |  | 97 |
| T2D16 | ● | ● | ● | ● | ● | ● | ● | ● | ● | ● | ○ | ● | ● | ● | ● | ● | ● | ● | ● | ● | ● | ● | ● | ● | ● | ● | ● | ○ | ○ | ○ | ● | ● | ● | ○ | **T** | **T** |  |  |  | 86 |
| T2D17 | ● | ● | ● | ● | ● | ● | ● | ● | ● | ● | ● | ● | ● | ● | ● | ● | ● | ● | ● | ● | ● | ● | ● | ● | ● | ● | ● | ● | ● | ● | ○ | ● | ● | **T** | ● | ● |  |  |  | 97 |
| T2D18 | ● | ● | ● | ● | ● | ● | ● | ● | ● | ● | ● | ● | ● | ● | ● | ● | ● | ● | ● | ● | ● | ● | ● | ● | ● | ● | ● | ● | ● | ● | ● | ○ | ● | **T** | ○ | ● |  |  |  | 94 |

Supplementary Table S4 All training sessions for each participant. Colour indicates the average percentage of heart rate reserve reached during the session (●>65%, ●60–65%, ●<60%, ●=no heart rate data available despite participation in a session) and filling indicates the length of the training session in percentage of the planned 45 minutes (○<25%, ◔25–50%, ◑50–75%, ◕75–95%, ●>95%). T=test day performing cycling tests (fat oxidation and *V̇*O_2peak_ test). CON=control. T2D=type 2 diabetes. Attnd.= the percentage of the planned 36 sessions (including the cycling test day) the participant attended, regardless of the duration and intensity of the session. Two participants had one extra training session to compensate for a cancelled training session. One participant had three extra training sessions due to illness on the last test day. One participant completed the post cycling test day two times due to technical issues.

| Supplementary Table S5 | | | | | |
| --- | --- | --- | --- | --- | --- |
|  | **Week 1-3** | **Week 4-6** | **Week 7-9** | **Week 10-12** | **Total average** |
| **HR (beats/min)** |  |  |  |  |  |
| CON | 139 ± 10 | 140 ± 9 | 139 ± 8 | 140 ± 7 | 140 ± 8 |
| T2D | 141 ± 8 | 140 ± 8 | 142 ± 7 | 143 ± 7 | 141 ± 8 |
| **HRR (%)** |  |  |  |  |  |
| CON | 69 ± 8 | 70 ± 9 | 70 ± 7 | 70 ± 7 | 70 ± 8 |
| T2D | 70 ± 6 | 70 ± 8 | 71 ± 6 | 72 ± 6 | 71 ± 6 |
| **Workload (watt)** |  |  |  |  |  |
| CON | 126 ± 34 | 140 ± 38 | 150 ± 40 | 152 ± 40 | 142 ± 37 |
| T2D | 104 ± 35 | 118 ± 38 | 129 ± 41 | 135 ± 46 | 121 ± 40 |

**Table S5**. Average heart rate during both the cycling and rowing sessions, and workload during the cycling sessions. Abbreviations: CON=control. T2D=type 2 diabetes. HR=heart rate. HRR=% of heart rate reserve.

**Fig. S1**. TREND flow diagram

Allocated to intervention: 12-week endurance training

T2D group (n = 20)

- Completed (n = 15)

- Did not complete (n = 5)

Control group (n = 17)

- Completed (n = 14)

- Did not complete (n = 3)

Follow-up and analysis:

T2D group analyzed (n = 15)

Control group analyzed (n = 14)

Assessed for eligibility (n = 300)

Excluded (n = 263):

- Not meeting criteria (n = 167)

- Declined (n = 60)

- Other (n = 36)

Enrolled (n = 37)

T2D group (n = 20)

Control group (n = 17)

Supplementary Fig. S2

**Fig. S2.** Progression of absolute *V̇*O_2peak_ throughout the intervention period with tests performed every three weeks. Abbreviations: CON=control. T2D=type 2 diabetes. Wk=week. ** p<0.01.

Supplementary Fig. S3

**Fig. S3** (a) Forearm blood flow, (b) glucose extraction, and (c) glucose uptake in the basal state and during a hyperinsulinaemic clamp before (pre) and after (post) 12 weeks of endurance training in individuals with type 2 diabetes (T2D) and a control group. B=basal. 1=clamp step 1. 2=clamp step 2. There was a main effect of insulin on all three variables (shown in each figure).

Supplementary Fig. S4

**Fig S4.** Basal and insulin-stimulated glucoregulatory hormones and metabolites in plasma. T2D=type 2 diabetes. B=basal. 1=clamp step 1. 2=clamp step 2. Statistically significant effects of group, training, insulin, and/or interaction are denoted in each subfigure. For insulin, post hoc analysis showed a significant effect of insulin infusion between all steps (p<0.001) but no differences between the groups or before and after training within a clamp step. For ketone bodies, post hoc analysis showed significant differences between the groups during basal conditions (p=0.01) and clamp step 1 (p<0.001) before training, as well as during clamp step 2 both before (p=0.007) and after (p=0.002) the training. The only significant training effect was a decrease in the diabetes group during clamp step 1 (p=0.03).The interaction reported is the three-way group x training x insulin interaction.
